# Accuracy and Repeatability of the Gait Analysis by the WalkinSense System

**DOI:** 10.1155/2014/348659

**Published:** 2014-02-20

**Authors:** Marcelo P. de Castro, Marco Meucci, Denise P. Soares, Pedro Fonseca, Márcio Borgonovo-Santos, Filipa Sousa, Leandro Machado, João Paulo Vilas-Boas

**Affiliations:** ^1^Centre of Research, Education, Innovation and Intervention in Sport, Faculty of Sports, University of Porto, Rua Dr. Plácido Costa 91, 4200 450 Porto, Portugal; ^2^Department of Physiotherapy, Activity and Human Movement Study Centre, School of Allied Health Science, Polytechnic Institute of Porto, Rua Valente Perfeito 322, 4400-330 Vila Nova de Gaia, Portugal; ^3^Porto Biomechanics Laboratory, University of Porto, Rua Dr. Plácido Costa 91, 4200 450 Porto, Portugal; ^4^University of Rome “Foro Italico,” Piazza Lauro de Bosis 6, 00135 Roma, Italy

## Abstract

WalkinSense is a new device designed to monitor walking. The aim of this study was to measure the accuracy and repeatability of the gait analysis performed by the WalkinSense system. Descriptions of values recorded by WalkinSense depicting typical gait in adults are also presented. A bench experiment using the Trublu calibration device was conducted to statically test the WalkinSense. Following this, a dynamic test was carried out overlapping the WalkinSense and the Pedar insoles in 40 healthy participants during walking. Pressure peak, pressure peak time, pressure-time integral, and mean pressure at eight-foot regions were calculated. In the bench experiments, the repeatability (i) among the WalkinSense sensors (within), (ii) between two WalkinSense devices, and (iii) between the WalkinSense and the Trublu devices was excellent. In the dynamic tests, the repeatability of the WalkinSense (i) between stances in the same trial (within-trial) and (ii) between trials was also excellent (ICC > 0.90). When the eight-foot regions were analyzed separately, the within-trial and between-trials repeatability was good-to-excellent in 88% (ICC > 0.80) of the data and fair in 11%. In short, the data suggest that the WalkinSense has good-to-excellent levels of accuracy and repeatability for plantar pressure variables.

## 1. Introduction

During human locomotion the foot acts passively, cushioning the impact forces, and actively, transferring the forces generated by the muscles to propel the body [1]. Thus, it is important to know the magnitude and behavior of ground reaction forces to prevent and treat injuries as well as to enhance human performance in sport. Biomechanical gait analysis is commonly performed by force plates that use three-dimensional force transducers to measure the three components (medial-lateral, anterior-posterior, and vertical) of ground reaction forces and torques [1]. For this reason, force plates are of enormous value for assessing human activity, such as walking, running, or jumping. However, this instrument does not directly provide any information about where these forces are being applied along the plantar surface of the foot. For this reason, new plantar pressure systems for quantitative gait force analysis have become increasingly popular.

The first documented described plantar pressure system, which was based on an air-filled chamber, was developed in the 19th century [2]. This method of measurement evolved to deformable materials that provided an ink impression or used optical methods for recording data [1]. At present, a wide range of systems that use electromechanical sensors allow for a more reliable plantar pressure evaluation. Among these, the capacitive, resistive, and piezoresistive sensors are the most frequently used for foot plantar pressure analysis. When compressed, they calculate the variations of the applied load, measuring the proportional change in voltage, conductance, or resistance, respectively [3–5]. These sensors can be used as either matrix measurements or discrete measurements [5]. Matrix measurements are made by arrays of sensors in the configuration of pressure plates for barefoot or in-shoe analyses that record the plantar pressure along the entire foot. Discrete measurements are performed using individual sensors (commonly up to eight sensors) placed at specific locations on the plantar foot surface (usually in-shoe). Compared to matrix measurements, discrete measurements are less complex but require an *a priori *decision about the regions of interest [5].

In-shoe plantar pressure systems (used to perform either discrete or matrix measurements) have been widely used by researchers and clinicians in the fields of clinical rehabilitation [6–8], ergonomics [9–11], and sport activities [12, 13]. Such systems allow monitoring of pressure in the interface between the plantar surface of the foot and the insole of a shoe during either static or dynamic activities, allowing measurements in real conditions without the limits of laboratorial setup [14, 15]. The operating principle behind each in-shoe system is generally the same: these systems use different sensors/insoles to collect and send pressure values to a hub, usually attached to the lateral malleolus or pelvic girdle, which records data in a memory card or transfers them in real-time to a computer by cable, Bluetooth, or other wireless means.

Before using a new device, validation studies against gold-standard instruments are conducted to determine the accuracy and repeatability of the system [14]. Accuracy is defined as the difference between the value of a known quantity and the value measured by the tested device [9, 16]. This device attribute is also referred to in the literature as “validity” [17, 18]. In the gait analysis field, calibration benches or systems considered to be gold standards are used to determine how accurate or valid the tested devices are. Another very important device feature is its repeatability, which is defined as the difference between two or more measurements performed by the same instrument under identical testing conditions [16, 19–21]. In gait studies of measurement error, many authors have also used the term “reliability” to refer to this feature [18, 20, 22–26]. In some studies the terms “repeatability” and “reliability” have been used indiscriminately [20, 21, 25]. In a repeatability study, variability in measurements made on the same subject can be ascribed only to errors in the measurement process itself. When gait analysis devices are assessed, the repeatability between stance phases (within-trial repeatability), between trials (between-trial repeatability), and between days (between-day repeatability) is commonly analyzed. One of the in-shoe plantar pressure devices most frequently used by clinicians and researchers is the Pedar in-shoe system (Novel GmbH, Munich, Germany). This system has been demonstrated to be accurate [9] and has shown excellent between-trial [15] and between-day [20, 26] repeatability. The knowledge of such device attributes (accuracy and repeatability) is of utmost importance before using it in clinical contexts.

WalkinSense (Kinematix SA, formerly Tomorrow Options, Sheffield, UK) is a user-friendly device designed for in-shoe monitoring and long-term storage of plantar pressure and spatial-temporal parameters during locomotion, such as gait speed, distance traveled, and stride length and frequency, without the need for a standardized calibration and the constraints of a laboratorial setup. One preliminary study [27] has already explored the repeatability of the plantar pressures recorded by the WalkinSense. The system was found to be as repeatable as other plantar pressure measurement systems (i.e., F-scan and Pedar). However, the authors assessed only three subjects and no statistical procedure was performed. The authors [27] highlighted the need for further investigations to truly understand how accurate and repeatable the plantar pressures measured by WalkinSense are. Another preliminary study assessed the spatial-temporal parameters of the WalkinSense [28] in a small sample of 15 participants and found good accuracy and repeatability for these parameters.

In short, there is very little information about how accurate the plantar pressures acquired by WalkinSense are, and it is not clear how repeatable the acquisition of these parameters is. This lack of information becomes a barrier for using the device in research and clinical contexts. Thus, the aim of this study was to measure the accuracy and repeatability of the gait analysis performed by the WalkinSense system. Values recorded by WalkinSense measuring typical gait in an adult population are also presented. We hypothesized that the plantar pressure parameters recorded by WalkinSense would be accurate and repeatable.

## 2. Methods

### 2.1. Participants

Forty volunteering university students (20 males and 20 females, with mean age of 21.6 ± 3.4, weight 67.2 ± 11.6 kg, and height 170.6 ± 0.9 cm) were recruited. All subjects were healthy and capable of ambulating independently. The exclusion criteria were any pain or difficulty with independent gait, disabilities that could affect natural gait (musculoskeletal, visual, or hearing impairments), and necessity of walking aids. This study was approved by the local ethical committee, and all participants freely gave their written consent to participate after being informed about the study procedures.

### 2.2. Equipment and Data Collection

**Figure 1:**
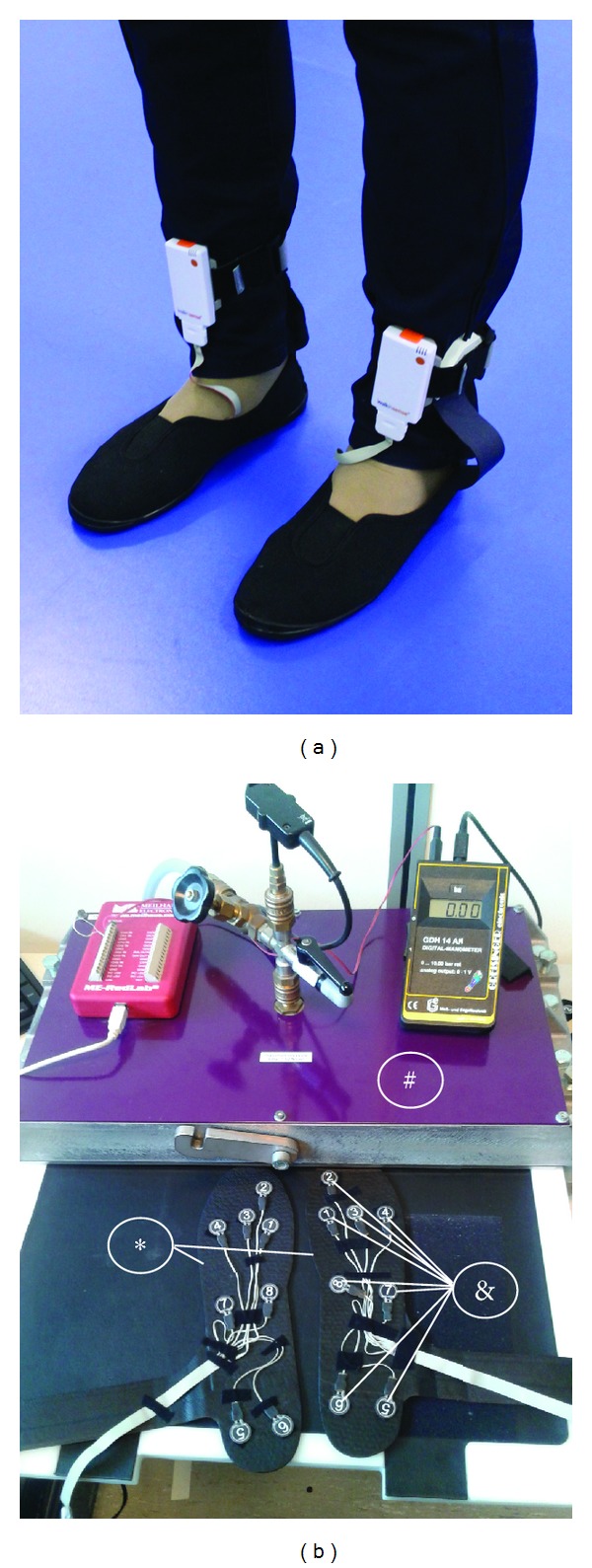
(a) WalkinSense and Pedar attached on one of the participants during data collection. (b) Experimental setup of the bench experiment: (&) position of the eight WalkinSense sensors on (∗) the Pedar insoles prior to insertion in (#) the Trublu calibration device.

The WalkinSense (weight: 68 g, length: 78 mm, width: 48 mm, and thickness: 18 mm) is a CE mark Class I electronic medical device designed to dynamically monitor human lower limb activity (Figure 1(a)). The device contains a micro-electromechanical system (MEMS) triaxial accelerometer and one gyroscope and is connected to a net of eight force-sensing piezoresistors (weight: 5 g, size: 1.8 cm^2^) used for foot pressure measurements, which can be freely positioned under or over any insole. This device operates at a sampling frequency of 100 Hz in two modes: offline mode, where data are stored to an SD memory card, and real-time mode, where data are communicated to a PC through Bluetooth technology.

Data collection was carried out into two phases: first, a gold-standard bench-testing comparison was conducted with the WalkinSense and the Trublu calibration device; second, a dynamic experimental test compared the WalkinSense with the Pedar during gait.

#### 2.2.1. Bench Experiment (Trublu)

During the bench testing two WalkinSense devices were assessed. Each one of the eight sensors of the WalkinSense nets were randomly positioned under an insole with 2 mm of thickness (provided by the manufacturer) and attached by adhesive Velcro straps. Next, the insoles were positioned into the Trublu, in which ten levels of pressure were sequentially applied during 10 seconds on each one: 0.00, 23.54, 49.03, 73.55, 99.05, 146.12, 199.07, 294.20, 391.29, and 492.29 kPa. Following this, the WalkinSense and the Pedar were assessed together (the WalkinSense nets were positioned under the Pedar insoles, Figure 1(b)), only to determine whether the two systems would work well jointly.

#### 2.2.2. Dynamic Experiment

For the dynamic experiment, the Pedar system (weight: 400 g, length: 150 mm, width: 100 mm, and thickness: 40 mm) was used. The Pedar records in-shoe plantar pressures through 99 capacitive pressure-sensitive sensors with an area of *≅*1.5 cm^2^ (depending on the size of the insole) and a sampling frequency of 100 Hz.

**Figure 2:**
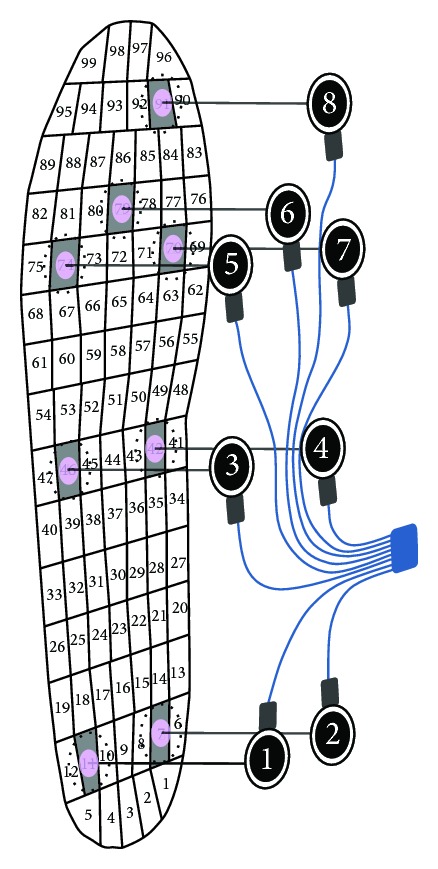
Position of the WalkinSense sensors at the Pedar insole.

The dynamic experimental protocol consisted of recording the plantar pressure during gait simultaneously, with the WalkinSense and the Pedar overlapped. Thus, we warranted that both systems were measuring exactly the same event. The bench tests suggested that there is no interference between the systems and they work well jointly. Before data collection, the Pedar insoles were checked by the Trublu calibration device in order to verify the performance of all sensors. The eight sensors of the WalkinSense were positioned under the Pedar insole (Figure 2) in correspondence with the main-reference foot areas, as proposed by and adapted from other studies [7, 10].

The centroid for positioning each of the WalkinSense sensors on the Pedar insole was manually identified by pressing a stick with the same area of the sensor on the insole corresponding to the selected regions. The activity of the Pedar sensors was controlled on a computer screen, and when only the aimed sensor was active, the region was marked. Following this, the WalkinSense sensors were attached to the insole using adhesive Velcro straps. This procedure was repeated for all pairs of Pedar insoles. The insoles were put into a neutral pair of shoes (ballet sneakers). Then, the participants stood in an upright position and their weight and height were recorded by a force plate (Bertec Corporation, Columbus, OH, USA) and a stadiometer (Seca, Birmingham, United Kingdom). The participants familiarized themselves with the experimental setup by walking freely over a 12-meter walkway at a pace of 100 steps per minute marked by electronic metronome software (Metronome Beat, Andy Stone). Following the familiarization, participants performed a variable number of trials, and three valid ones were used for further analysis. In each trial, about 12 steps were recorded and only the central four stance phases (two with each foot) were used in the statistical analysis. This procedure was adopted to avoid the effects of acceleration in the movement.

### 2.3. Data Analysis

Data from the Pedar were recorded by the Pedar-X software (Novel GmbH, Munich, Germany) and data from the WalkinSense using the WalkinSense software (Kinematix SA, Sheffield, UK). The sensor pressure values from both systems were exported and then analyzed by MATLAB 7.0 software (MathWorks, Massachusetts, USA) through an appropriate program for data processing and variable calculation. Each step of all trials was considered as one sample during the statistical analysis to avoid hiding differences between the instruments and within or between trials that could be caused by averaged values.

#### 2.3.1. Bench Experiment

At each of the ten load levels applied by the Trublu, we analyzed the 100 central frames (from 1000 recorded frames—10 seconds of data recording) taken from the two WalkinSense systems. The loads applied by the Trublu were also recorded for later use as reference values to calculate the accuracy of the WalkinSense systems.

#### 2.3.2. Dynamic Experiment

The following anatomical regions were studied: great toe (G_Toe_); medial, central, and lateral forefoot (FF_Med_, FF_Ct_, and FF_Lat_, resp.); medial and lateral midfoot (MF_Med_ and MF_Lat_, resp.); and medial and lateral rearfoot (RF_Med_ and RF_Lat_, resp.). For each WalkinSense sensor and the respective Pedar sensor, four dependent variables were calculated: peak pressure (*P*_Peak_, in kPa), defined as the highest value displayed by the sensor along the stance phase; peak pressure time (*P*_Time_, in % of the stance phase), defined as the instant correspondent to the *P*_Peak_; mean pressure (*P*_Mean_, in kPa), defined as the mean pressure during the stance phase; and the pressure-time integral (*P*_Integral_, in kPa s), defined as the integral along the stance phase.

The gait analysis conducted used the mid-gait method, as this method aptly represents a normal walk [29], and three trials were performed in order to provide a consistent mean [30]. Subjects wore standardized shoes and adopted a controlled gait cadence, since footwear and walking speed have been shown to influence plantar pressures during gait [31]. Gender differences were not considered, as a previous article reported no gender influence on *P*_Peak_ and *P*_Integral_ parameters [32].

### 2.4. Statistical Analysis

Statistical analysis was performed using SPSS statistics v.20 software (IBM SPSS, Chicago, USA) and Statistica v.8 software (Statsoft, Tulsa, USA). We considered the Two-Way Mixed Model (Type: consistency) intraclass correlation coefficient (ICC) ≤0.69 as poor, 0.70–0.79 as fair, 0.80–0.89 as good, and ≥0.90 as excellent [33]. All ICCs were calculated intraexaminers. The 95% confidence intervals (CI_95%_) were calculated with the ICC and the absolute and percentage differences to verify the uncertainty of these differences [34].

#### 2.4.1. Bench Experiment

*(1) Repeatability.* The within-WalkinSense (sensor versus sensor from the same WalkinSense net) and between-WalkinSense (8 sensors versus 8 sensors from two WalkinSense systems) repeatability was verified by the ICC.

*(2) Accuracy.* The relation (accuracy) between the applied load (Trublu) and WalkinSense was verified by the (i) ICC, (ii) Pearson correlation coefficient, and (iii) absolute (Trublu − WalkinSense) and percentage [(Trublu − WalkinSense) × 100/Trublu] difference analyses. Negative values indicate that the WalkinSense showed values higher than the Trublu, while positive values indicate that the WalkinSense showed values lower than the Trublu.

#### 2.4.2. Dynamic Experiments

*(1) Repeatability.* The overall (considering all regions together) and regional within-trial (first right step versus second right step and first left step versus second left step) and between-trial (four steps from the first trial versus second trial versus third trial) repeatability of the WalkinSense was verified by the ICC.

*(2) Accuracy.* The relation (accuracy) between the WalkinSense and the Pedar records was verified by (i) the ICC (overall and regional), (ii) the overall Person correlation coefficient, and (iii) the overall and regional absolute (Pedar − WalkinSense) and percentage [(Pedar − WalkinSense) × 100/Pedar] difference analyses. Negative values indicate that the WalkinSense showed values higher than the Pedar, while positive values indicate that the WalkinSense showed values lower than the Pedar.

*(3) Description of Values.* The descriptive statistics include mean and standard deviation for each parameter of the WalkinSense and the Pedar for each foot region.

## 3. Results

### 3.1. Bench Experiment

#### 3.1.1. Repeatability

**Figure 3:**
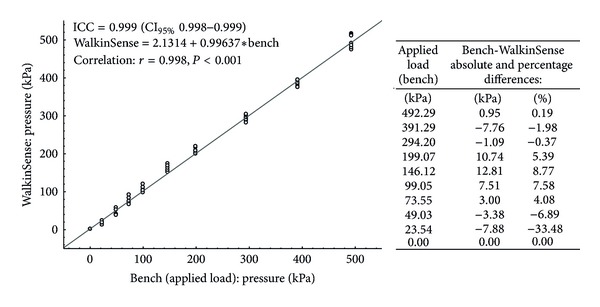
Bench experiment: relation between the applied loads and WalkinSense records at the 10 levels of load. ICC: intraclass correlation coefficient. At the second and third column of the table, positive values indicate greater values in the WalkinSense system and negative values indicate lower values in the WalkinSense.

The within-WalkinSense ICC was 0.999 (CI_95%_ 0.998 to 0.999) (Figure 3) and the between-WalkinSense ICC was 0.993 (CI_95%_ 0.990 to 0.995).

#### 3.1.2. Accuracy

An excellent ICC and high and statistically significant (*P* < 0.001) level of correlation between the applied loads (Trublu) and WalkinSense records were observed (Figure 3). The absolute differences ranged from −7.88 to 12.81 kPa along the different applied loads. The percentage differences were lower than 9% for nine out of the ten loads applied. At the first load applied (25.54 kPa), the WalkinSense showed pressure values 33.48% lower than the bench. The heaviest applied loads (>294 kPa) showed the smallest differences (<2%).

### 3.2. Dynamic Experiments

#### 3.2.1. Repeatability

Excellent overall within-trial and between-trial repeatability was found in the four dependent variables, and all of the ICC CIs_95%_ were smaller than 0.02 (Table 1).

The regional within-trial and between-trial ICCs for the *P*_Peak_ were excellent in four (RF_Lat_, RF_Med_, FF_Ct_, and G_Toe_), good in two (FF_Lat_ and FF_Med_), and fair in one (MF_Lat_) out of the seven analyzed regions (Table 2). For the *P*_Time_, the regional within-trial ICCs were excellent (G_Toe_), good (FF_Lat_), and poor (MF_Lat_) in one region each and fair in four regions; and the between-trial ICCs were good in five regions and excellent (G_Toe_) and fair (MF_Lat_) in one region each. All within-trial and between-trial ICCs for *P*_Integral_ and *P*_Mean_ were good or excellent (Table 2).

#### 3.2.2. Accuracy

Analyzing all regions together (overall), the WalkinSense showed lower values for *P*_Peak_ (6.2%), *P*_Integral_ (14.1%), and *P*_Mean_ (13.2%) compared to the Pedar. The *P*_Peak_ occurred slightly later (3.3%) in the WalkinSense compared to the Pedar (Table 3). The overall ICCs (WalkinSense versus Pedar) were higher than 0.95 for all variables (Table 3). The Pearson coefficient indicated correlations statistically significant for all variables (Table 3).

The regional percentage differences between the systems for *P*_Peak_ ranged from 3.6% at the RF_Med_ to 16.4% at the MF_Lat_. The percentage differences for the *P*_Time_ were similar among the regions, ranging from 2.3% to 4.3%. In the *P*_Integral_ and *P*_Mean_, the highest differences were observed at the MF_Lat_ (*≅*30% for both *P*_Integral_ and *P*_Mean_) and the lowest differences in the RF_Med_ (7.7% for *P*_Integral_ and 6.3% for *P*_Mean_) (Table 4).

In seven out of the eight regions, lower values were observed in the WalkinSense (negative percentage differences) for the *P*_Time_, *P*_Integral_, and *P*_Mean_ variables. Only at the FF_Ct_ did the WalkinSense show higher values than the Pedar. The range of the CIs_95%_ of the percentage differences for the *P*_Peak_ was *≅*3.5%; for the *P*_Time_, it was *≅*1.5%; and for the *P*_Integral_ and *P*_Mean_, it was *≅*4.5% (Table 4).

Twenty-six out of the 28 regional ICCs (Pedar and WalkinSense) were greater than 0.9, and their 95% confidence interval ranged between 0.2 and 0.35. The remaining two ICCs (out of the 28) were 0.86 for the *P*_Peak_ at the RF_Lat_ and 0.84 for the *P*_Time_ at the FF_Ct_.

#### 3.2.3. Description of Values

Both systems showed the highest and lowest *P*_Peak_, *P*_Integral_, and *P*_Mean_ values at the FF_Ct_ and MF_Med_, respectively. Also, the earliest and latest *P*_Time_ occurred at the same regions in both systems (RF_Lat_ and G_Toe_, resp.) (Table 5).

In most trials of both systems, there was no pressure at the MF_Med_. In 472 out of 480 of the WalkinSense stance phases analyzed (four stance phases × three trails × 40 participants) and 436 out of 480 of the Pedar stance phases, the MF_Med_ was not loaded. The four highest values for *P*_Peak_ (WalkinSense: 42.2, 32.4, 16.7, and 12.7 kPa; Pedar: 77.5, 62.5, 57.5, and 52.2 kPa), *P*_Integral_ (WalkinSense: 6.2, 4.8, 2.5, and 0.8 kPa s; Pedar: 15.6, 13.0, 12.7, and 9.1 kPa s), and *P*_Mean_ (WalkinSense: 10.9, 9.7, 4.3, and 1.4 kPa; Pedar: 21.7, 19.1, 18.1, and 13.2 kPa) occurred at the same trials in both systems.

## 4. Discussion

The present study aimed to measure the accuracy, repeatability, and description of values of the plantar pressure parameters of the WalkinSense system. For this purpose, two experiments were carried out: a bench experiment, in which the WalkinSense records were compared to a gold-standard bench test (Trublu), and a dynamic experiment, in which the WalkinSense was compared to one of the most commonly used in-shoe plantar pressure systems (Pedar) in gait analyses. While the majority of the measurement error studies only assess the repeatability or accuracy of the devices using static-bench [9, 17, 35] or dynamic [19–26, 36] experiments separately, the present study focused on a wider approach for statically and dynamically assessing the accuracy and repeatability of a device.

### 4.1. Bench Experiment

Our first aim was to assess repeatability of the eight WalkinSense sensors from a single net as well as the repeatability of a couple of nets. Secondly, we wanted to verify the accuracy of the measurements when compared to a gold-standard device. Results show an excellent repeatability (ICC > 0.999) of the measurements for both the single net and pair of nets, together with an excellent overall repeatability and a high Pearson correlation coefficient between the WalkinSense and Trublu systems. However, further analysis reported that the absolute and the percentage differences varied with the applied loads. In fact, the highest percentage differences, −33% and −7%, were observed at the lowest pressures, 24 and 49 kPa, applied. Hsiao et al. [9] reported similar results in a previous study where the accuracy of the Pedar and F-scan (Tekscan, South Boston, USA) systems were analyzed by bench tests. They found a low accuracy at the lowest pressures in both systems and a gradual reduction of the percentage difference at the higher loads. The Pedar showed percentage differences between −57.2% and 1.3% when pressures between 12 and 59 kPa were applied; the F-scan reported a similar trend with percentage differences between 19.4% and 27.9% for loads between 5 and 41 kPa [9]. In our study, on the other hand, we observed the lowest percentage differences, equal to 0.4% and 0.2%, at the highest load magnitudes (*≅*300 and 500 kPa). In the same way, when the Pedar and F-scan systems were loaded with 300 and 500 kPa by Hsiao et al. [9], low differences, equal to 5.2% and 3.6% and 1.2% and −11%, respectively, were observed. As the thickness of the contact surface in which the sensors are placed decreases their sensitivity at low-pressure ranges (from 10 to 80 kPa) [37], the insole in which the WalkinSense sensors were placed, with its 2 mm thickness, may play an important role in explaining these higher percentage differences found at the lowest applied pressures.

### 4.2. Dynamic Experiment

Excellent overall within- and between-trial repeatability was found for all dependent variables (*P*_Peak_, *P*_Time_, *P*_Mean_, and *P*_Integral_) in this study. However, the ICCs varied among regions. For both *P*_Peak_ and *P*_Integral_, six out of the seven regions (disregarding the MF_Med_, which was not analyzed) reported a good-to-excellent repeatability, while only one, the MF_Lat_, reported fair values. Similarly, in a study by Kernozec et al. [20], where the repeatability of the Pedar system was assessed by the coefficient of variation, different results were obtained among the regions. As in our study, the authors [20] also found the midfoot (which was considered as a unique region) to be one of the least repeatable regions. In another study in which the between-day repeatability of a pressure plate (EMED) was assessed in ten-foot regions, Gurney et al. [24] reported that the *P*_Peak_ repeatability was poor-to-fair in four regions and good-to-excellent in six, while the *P*_Integral_ repeatability was poor-to-fair in three regions and good-to-excellent in the remaining seven [24].

In our study, overall high degrees of agreement (ICCs > 0.95) were found comparing gait parameters of the WalkinSense and the Pedar. The overall percentage differences indicate that the WalkinSense showed lower *P*_Peak_ (*≅*6%), *P*_Integral_ (*≅*14%), and *P*_Mean_ (*≅*13%) and the *P*_Time_ slightly later (*≅*3%) compared to the Pedar. The regional ICCs between the systems were excellent for almost all regions. When considering the regional percentage differences, *P*_Integral_ and *P*_Mean_ reported the highest differences: *≅*30% in the MF_Lat_ and *≅*24% in the FF_Lat_. Considering the differences between the systems (kind of sensor, sensor area, and layout), we may consider these differences as acceptable.

In a preliminary study, Healy et al. [27] assessed three subjects' walking using the WalkinSense and the F-scan (Tekscan Inc., Boston, USA) systems in two different days. The WalkinSense showed a similar level of repeatability when compared to other plantar pressure measurement systems. However, the conclusions were mostly based on subjective analyses; the authors did not provide any measure of variance of the values nor did they perform any statistical procedure. Therefore, it is difficult to compare their results [27] with those found in the present study. Nevertheless, our findings seem to be in agreement with those reported by Healy and colleagues in their preliminary study [27].

In short, in the dynamic experiment (gait analysis) from the present study, the overall within-trial and between-trial repeatability were excellent and the regional within-trial and between-trial repeatability were mostly good-to-excellent (only in one region was repeatability fair). The overall accuracy (ICCs: WalkinSense versus Pedar) showed ICCs higher than 0.95 and Pearson coefficient with correlations statistically significant for all variables and for the regional accuracy ICCs higher than 0.84 were observed for all variables.

### 4.3. Description of Values

All the *P*_Peak_ values found in the present study fell in the reference range previously proposed for healthy subjects [19]. The *P*_Integral_ magnitudes and the sequence of the *P*_Time_ along the regions were similar to those presented by Putti et al. [19].

In the present study, the highest *P*_Peak_ was in the FF_Ct_ (331 kPa), followed by the RF_Med_ (275 kPa) and the RF_Lat_ (257 kPa), while in the aforementioned study [19] the highest *P*_Peak_ occurred in the G_Toe_ (280 kPa), followed by the rearfoot (which was considered as one region, 264 kPa) and forefoot (metatarsal heads I and II, *≅*247 kPa). In another study assessing a healthy population [36], the highest *P*_Peak_ was found in the forefoot (metatarsal heads II—361 kPa and III—330 kPa), followed by the G_Toe_ (321 kPa) and the rearfoot (313 kPa), using a pressure plate. The differences among these studies could be attributed to external variables such as the use of different shoes (neutral shoes versus running shoes) or different reference systems (in-shoe pressure system versus pressure plate). In the present study, we opted to use an in-shoe pressure system inside of neutral shoes in order to record gait values during a condition as similar as possible to those found in daily life.

### 4.4. Limitations

This study presents some limitations, such as (i) the standardized position of the WalkinSense sensors for the four pairs of Pedar insoles that did not necessarily correspond to the point of maximal pressure for all subjects; (ii) the differences between the WalkinSense and the Pedar (i.e., layout, sensor area, and kind); and (iii) the description of values provided in this study that can only be considered for the proposed arrangement of the sensors.

## 5. Conclusion

The WalkinSense showed good-to-excellent levels of accuracy and repeatability for plantar pressure variables during static-bench and dynamic gait analysis. Four plantar pressure parameters in healthy adults were presented and can be used as standard values while using this device. Further investigation of gait analysis and of the long-term accuracy and repeatability, the between-day and interexaminer repeatability, and the accuracy and repeatability of the spatial-temporal parameters of the WalkinSense are needed.

## Figures and Tables

**Table 1:** WalkinSense overall (all regions together) within- and between-trials intraclass correlation coefficients (ICC) for all measurements during gait.

Variables	Within-trial			Between-trial		
	ICC	CI_95%_		ICC	CI_95%_	
*P*_Peak_	0.972	0.969	0.975	0.979	0.977	0.981
*P*_Time_	0.987	0.986	0.988	0.915	0.904	0.925
*P*_Mean_	0.940	0.933	0.946	0.993	0.992	0.994
*P*_Integral_	0.938	0.931	0.944	0.965	0.960	0.969

*P*_Peak_: peak pressure; *P*_Time_: pressure time; *P*_Mean_: mean pressure; *P*_Integral_: pressure-time integral.

**Table 2:** WalkinSense regional within- and between-trial intraclass correlation coefficients (ICC) during gait.

Variable	Region	Within-trial			Between-trial		
		ICC	CI_95%_		ICC	CI_95%_	
Peak pressure	RF_Lat_	0.978	0.971	0.983	0.971	0.961	0.979
	RF_Med_	0.964	0.953	0.973	0.961	0.948	0.972
	MF_Lat_	0.763	0.688	0.821	0.794	0.713	0.855
	MF_Med_	&	&	&	&	&	&
	FF_Lat_	0.868	0.826	0.900	0.896	0.858	0.926
	FF_Ct_	0.949	0.932	0.961	0.956	0.940	0.968
	FF_Med_	0.834	0.779	0.875	0.858	0.802	0.900
	G_Toe_	0.928	0.905	0.946	0.966	0.954	0.976

Peak pressure time	RF_Lat_	0.718	0.630	0.785	0.859	0.808	0.897
	RF_Med_	0.763	0.684	0.823	0.826	0.758	0.877
	MF_Lat_	0.665	0.554	0.749	0.763	0.669	0.834
	MF_Med_	&	&	&	&	&	&
	FF_Lat_	0.873	0.831	0.904	0.857	0.801	0.900
	FF_Ct_	0.786	0.717	0.838	0.808	0.736	0.863
	FF_Med_	0.748	0.664	0.811	0.843	0.780	0.891
	G_Toe_	0.900	0.867	0.924	0.946	0.926	0.962

Pressure-time integral	RF_Lat_	0.959	0.946	0.969	0.960	0.946	0.971
	RF_Med_	0.908	0.879	0.930	0.928	0.901	0.948
	MF_Lat_	0.848	0.798	0.885	0.873	0.825	0.910
	MF_Med_	&	&	&	&	&	&
	FF_Lat_	0.890	0.855	0.917	0.912	0.879	0.937
	FF_Ct_	0.960	0.948	0.970	0.965	0.953	0.975
	FF_Med_	0.810	0.746	0.857	0.835	0.769	0.885
	G_Toe_	0.882	0.843	0.911	0.915	0.882	0.939

Mean pressure	RF_Lat_	0.965	0.955	0.974	0.961	0.947	0.972
	RF_Med_	0.903	0.871	0.926	0.928	0.902	0.949
	MF_Lat_	0.817	0.758	0.862	0.867	0.814	0.906
	MF_Med_	&	&	&	&	&	&
	FF_Lat_	0.884	0.847	0.912	0.915	0.883	0.939
	FF_Ct_	0.959	0.947	0.969	0.962	0.948	0.973
	FF_Med_	0.816	0.755	0.862	0.840	0.774	0.889
	G_Toe_	0.890	0.853	0.917	0.916	0.885	0.941

RF_Lat_: lateral rearfoot; RF_Med_: medial rearfoot; MF_Lat_: lateral midfoot; MF_Med_: medial midfoot; FF_Lat_: lateral forefoot; FF_Ct_: central forefoot; MF_Med_: medial forefoot; G_Toe_: great toe; CI_95%_: 95% confidence interval. &: as the midfoot region was loaded only in *≅*5% of the trials, the percentage difference and ICC were not calculated for this region.

**Table 3:** Percentage difference, intraclass correlation coefficient (ICC), and Pearson correlation coefficient (CC) between Pedar and WalkinSense during gait.

	Percentage differences (%)			ICC			Pearson CC	
	Average difference	CI_95%_		ICC	CI_95%_		*r*	*p*
*P*_Peak_	−6.2	−6.8	−5.6	0.973	0.971	0.975	0.869	<0.001
*P*_Time_	3.4	3.1	3.6	0.997	0.997	0.997	0.973	<0.001
*P*_Mean_	−14.1	−14.9	−13.2	0.955	0.952	0.958	0.872	<0.001
*P*_Integral_	−13.2	−14.1	−12.4	0.956	0.953	0.959	0.872	<0.001

*P*_Peak_: peak pressure; *P*_Time_: pressure time; *P*_Mean_: mean pressure; *P*_Integral_: pressure-time integral.

**Table 4:** Percentage differences and intraclass correlation coefficient (ICC) between Pedar and WalkinSense for each foot region during gait.

Variable	Region	Percentage differences (%)			ICC	CI_95%_	
		Average difference	CI_95%_				
Peak pressure	RF_Lat_	−10.8	−12.4	−9.1	0.859	0.827	0.885
	RF_Med_	−3.6	−5.0	−2.2	0.958	0.949	0.965
	MF_Lat_	−16.4	−18.1	−14.6	0.963	0.954	0.971
	MF_Med_	&	&	&	&	&	&
	FF_Lat_	−15.2	−16.7	−13.7	0.936	0.922	0.947
	FF_Ct_	−5.4	−6.9	−3.9	0.953	0.942	0.961
	FF_Med_	10.4	8.5	12.4	0.945	0.932	0.956
	G_Toe_	−7.9	−9.8	−6.1	0.953	0.942	0.961

Peak pressure time	RF_Lat_	4.3	3.1	5.5	0.974	0.967	0.980
	RF_Med_	3.4	2.2	4.5	0.982	0.977	0.985
	MF_Lat_	3.6	2.9	4.3	0.983	0.980	0.986
	MF_Med_	&	&	&	&	&	&
	FF_Lat_	3.7	3.3	4.1	0.964	0.956	0.971
	FF_Ct_	2.3	1.9	2.7	0.839	0.803	0.868
	FF_Med_	3.0	2.6	3.5	0.944	0.931	0.954
	G_Toe_	3.5	3.1	3.8	0.938	0.923	0.949

Pressure-time integral	RF_Lat_	−18.4	−20.7	−16.2	0.907	0.888	0.923
	RF_Med_	−7.7	−9.6	−5.8	0.949	0.937	0.958
	MF_Lat_	−31.2	−33.7	−28.8	0.937	0.923	0.948
	MF_Med_	&	&	&	&	&	&
	FF_Lat_	−24.7	−26.6	−22.8	0.915	0.897	0.929
	FF_Ct_	8.4	6.4	10.4	0.923	0.907	0.937
	FF_Med_	−12.2	−13.8	−10.6	0.961	0.952	0.968
	G_Toe_	−14.0	−15.8	−12.2	0.959	0.950	0.966

Mean pressure	RF_Lat_	−17.5	−19.8	−15.2	0.915	0.897	0.930
	RF_Med_	−6.3	−8.1	−4.4	0.947	0.936	0.957
	MF_Lat_	−30.1	−32.6	−27.7	0.942	0.927	0.953
	MF_Med_	&	&	&	&	&	&
	FF_Lat_	−23.7	−25.6	−21.8	0.913	0.895	0.929
	FF_Ct_	9.8	7.8	11.8	0.949	0.937	0.959
	FF_Med_	−11.0	−12.6	−9.4	0.959	0.950	0.967
	G_Toe_	−13.3	−15.1	−11.4	0.970	0.963	0.975

RF_Lat_: lateral rearfoot; RF_Med_: medial rearfoot; MF_Lat_: lateral midfoot; MF_Med_: medial midfoot; FF_Lat_: lateral forefoot; FF_Ct_: central forefoot; MF_Med_: medial forefoot; G_Toe_: great toe; CI_95%_: 95% confidence interval. &: as the midfoot region was loaded only in *≅*5% of the trials, the percentage difference and ICC were not calculated for this region.

**Table 5:** Description of values for the WalkinSense and Pedar during gait.

Variable	Region	Pedar		WalkinSense	
		Mean	SD	Mean	SD
Peak pressure (kPa)	RF_Lat_	291.0	73.0	257.2	71.6
	RF_Med_	285.3	95.8	274.7	96.3
	MF_Lat_	80.8	35.0	67.8	34.2
	MF_Med_	0.8	6.4	0.3	2.7
	FF_Lat_	250.0	86.7	211.9	80.8
	FF_Ct_	302.9	104.1	330.7	110.8
	FF_Med_	257.0	88.8	242.4	86.9
	G_Toe_	247.0	121.7	224.2	111.9

Peak pressure time (%Stance)	RF_Lat_	16.2	5.1	16.9	5.4
	RF_Med_	17.8	6.5	18.4	6.8
	MF_Lat_	48.2	11.5	49.6	11.3
	MF_Med_	26.7	8.7	33.5	13.3
	FF_Lat_	71.0	7.4	73.6	7.4
	FF_Ct_	76.9	4.1	78.6	3.6
	FF_Med_	72.3	6.7	74.3	6.4
	G_Toe_	80.6	6.0	83.3	5.6

Pressure-time integral (kPa·s)	RF_Lat_	70.5	26.6	58.1	27.8
	RF_Med_	70.7	27.0	65.6	28.9
	MF_Lat_	25.8	10.6	18.3	11.0
	MF_Med_	0.4	1.6	0.0	0.4
	FF_Lat_	82.5	26.0	63.5	27.9
	FF_Ct_	84.1	30.7	90.7	35.2
	FF_Med_	78.4	28.3	69.9	30.3
	G_Toe_	57.7	32.9	50.4	32.0

Mean pressure (kPa)	RF_Lat_	94.5	33.0	79.1	36.3
	RF_Med_	96.2	37.1	90.4	39.5
	MF_Lat_	34.5	14.4	24.7	14.3
	MF_Med_	0.5	2.3	0.1	0.7
	FF_Lat_	111.5	33.2	86.5	36.1
	FF_Ct_	112.9	39.4	123.2	44.9
	FF_Med_	106.1	37.4	95.7	40.4
	G_Toe_	78.3	43.5	69.0	42.8

RF_Lat_: lateral rearfoot; RF_Med_: medial rearfoot; MF_Lat_: lateral midfoot; MF_Med_: medial midfoot; FF_Lat_: lateral forefoot; FF_Ct_: central forefoot; MF_Med_: medial forefoot; G_Toe_: great toe; CI_95%_: 95% confidence interval; SD: standard deviation.
